# *FADS1 FADS2* Gene Cluster, PUFA Intake and Blood Lipids in Children: Results from the GINIplus and LISAplus Studies

**DOI:** 10.1371/journal.pone.0037780

**Published:** 2012-05-21

**Authors:** Marie Standl, Eva Lattka, Barbara Stach, Sibylle Koletzko, Carl-Peter Bauer, Andrea von Berg, Dietrich Berdel, Ursula Krämer, Beate Schaaf, Stefan Röder, Olf Herbarth, Anette Buyken, Tim Drogies, Joachim Thiery, Berthold Koletzko, Joachim Heinrich, for the GINIplus Study Group, and LISAplus Study Group

**Affiliations:** 1 Institute of Epidemiology I, Helmholtz Zentrum München – German Research Centre for Environmental Health, Neuherberg, Germany; 2 Research Unit of Molecular Epidemiology, Helmholtz Zentrum München – German Research Centre for Environmental Health, Neuherberg, Germany; 3 Faculty of Medicine, Institute of Laboratory Medicine, Clinical Chemistry, and Molecular Diagnostics, University Hospital Leipzig, Leipzig, Germany; 4 University of Munich Medical Centre, Dr. von Hauner Children's Hospital, Munich, Germany; 5 Department of Pediatrics, Technical University of Munich, Munich, Germany; 6 Department of Pediatrics, Marien-Hospital Wesel, Wesel, Germany; 7 IUF, Leibniz Institut für Umweltmedizinische Forschung at the University of Düsseldorf, Düsseldorf, Germany; 8 Medical Practice for Pediatrics, Bad Honnef, Germany; 9 Department for Environmental Immunology, Helmholtz Centre for Environmental Research – UFZ, Leipzig, Germany; 10 Faculty of Medicine, Environmental Medicine and Hygiene, University of Leipzig, Leipzig, Germany; 11 Research Institute of Child Nutrition, University of Bonn, Dortmund, Germany; Erasmus Medical Center, The Netherlands

## Abstract

**Background:**

Elevated cholesterol levels in children can be a risk factor for cardiovascular diseases in later life. In adults, it has been shown that blood lipid levels are strongly influenced by polymorphisms in the fatty acid desaturase (*FADS*) gene cluster in addition to nutritional and other exogenous and endogenous determinants. Our aim was to investigate whether lipid levels are determined by the *FADS* genotype already in children and whether this association interacts with dietary intake of n-3 fatty acids.

**Methods:**

The analysis was based on data of 2006 children from two German prospective birth cohort studies. Total cholesterol, HDL, LDL and triglycerides were measured at 10 years of age. Six single nucleotide polymorphisms (SNPs) of the *FADS* gene cluster were genotyped. Dietary n-3 fatty acid intake was assessed by food frequency questionnaire. Linear regression modeling was used to assess the association between lipid levels, n-3 fatty acid intake and *FADS* genotype.

**Results:**

Individuals carrying the homozygous minor allele had lower levels of total cholesterol [means ratio (MR) ranging from 0.96 (p = 0.0093) to 0.98 (p = 0.2949), depending on SNPs] and LDL [MR between 0.94 (p = 0.0179) and 0.97 (p = 0.2963)] compared to homozygous major allele carriers. Carriers of the heterozygous allele showed lower HDL levels [β between −0.04 (p = 0.0074) to −0.01 (p = 0.3318)] and higher triglyceride levels [MR ranging from 1.06 (p = 0.0065) to 1.07 (p = 0.0028)] compared to homozygous major allele carriers. A higher n-3 PUFA intake was associated with higher concentrations of total cholesterol, LDL, HDL and lower triglyceride levels, but these associations did not interact with the *FADS1 FADS2* genotype.

**Conclusion:**

Total cholesterol, HDL, LDL and triglyceride concentrations may be influenced by the *FADS1 FADS2* genotype already in 10 year old children. Genetically determined blood lipid levels during childhood might differentially predispose individuals to the development of cardiovascular diseases later in life.

## Introduction

Lipid concentrations in blood are associated with cardiovascular diseases [1]–[4]. Elevated cholesterol levels during childhood and adolescence increases the risk for cardiovascular diseases in adulthood. Moreover treatments which effectively lower cholesterol levels early in life have been shown to prevent disease manifestation in later life [5]–[7].

Among other factors, dietary polyunsaturated fatty acid (PUFA) intake can attenuate high blood lipid concentrations [8]–[10].

In addition to dietary influences, recent genome wide association studies have identified several genetic loci that are associated with blood lipid levels in adults [11]–[15]. Among the top hits for the determination of lipid levels are polymorphisms in the fatty acid desaturase (*FADS*) gene cluster. Lower total cholesterol [11], low-density lipoprotein (LDL) [11]–[13], high-density lipoprotein (HDL) [13], [14] and higher triglyceride levels [14], [15] are all associated with the minor alleles of the tested *FADS* polymorphisms. The genes *FADS1* and *FADS2*, which are both located in this gene cluster, encode the enzymes delta-5-desaturase and delta-6-desaturase, which are involved in the conversion of dietary n-3 and n-6 fatty acids to their longer chain metabolites [16]. Polymorphisms in these genes are associated with n-3 and n-6 fatty acid levels in blood and several tissues [17]–[23]. Carriers of the minor alleles exhibit increased levels of desaturase substrates and decreased levels of desaturase products. This trend may be the result of lower transcription levels or diminished enzyme conversion rates in individuals carrying the minor alleles [17].

The previously reported strong association of *FADS* polymorphisms with lipid levels in adults makes these genes good candidates for genetic association studies focused on lipid levels in children. Given the role of the gene products in the conversion of dietary fatty acids to biologically important longer chain polyunsaturated fatty acids, which in turn have been shown to influence lipid levels, interaction analysis between genetic polymorphisms and dietary intake of fatty acids for individual lipid levels is of interest.

In this study we investigated whether genetic variations of the *FADS* gene cluster already pre-determines total cholesterol, HDL, LDL and triglyceride levels in 10 year old children and whether genetic variation interacts with dietary intake of n-3 PUFAs to determine individual blood lipid levels.

## Results

Information on lipid concentrations in blood and *FADS1 FADS2* genotype was available for 2006 children (1288 (64%) children from the GINIplus study and 718 (36%) children from the LISAplus study). Information on n-3 PUFA intake was only available for 1697 of these children (1100 (65%) children from the GINIplus study and 597 (35%) children from the LISAplus study).

Basic characteristics of the study population are presented in Table 1. Total cholesterol, LDL and triglyceride concentrations were significantly higher in the GINIplus study, whereas HDL concentrations were higher in the LISAplus study.

**Table 1:** Basic characteristics of the study population.

	GINIplus (n = 1288)	LISAplus (n = 718)	Total (n = 2006)	p-value
	Median (25%-Qu.,75%-Qu.) or %			
**Gender [% male]**	49.0	55.6	51.3	0.0052^1^
**Age [weeks]**	531 (526,538)	529 (525,536)	530 (525,537)	0.0072^2^
**BMI**	17 (16,19)	17 (15,18)	17 (16,19)	<0.0001^2^
**Fasting blood samples [%]**	16.5	21.9	18.4	0.0032^1^
**n-3 PUFA intake [mg/MJ]**	0.14 (0.13,0.16)	0.14 (0.13,0.16)	0.14 (0.13,0.16)	0.5950^2^
**Total energy intake [MJ]**	8237 (6900,9907)	8198 (6654,9906)	8231 (6824,9906)	0.4012^2^
**Total cholesterol [mmol/L]**	4.81 (4.28,5.39)	4.75 (4.30,5.23)	4.79 (4.28,5.32)	0.0422^2^
**HDL [mmol/L]**	1.20 (1.02,1.41)	1.33 (1.15,1.50)	1.24 (1.06,1.44)	<0.0001^2^
**LDL [mmol/L]**	2.14 (1.73,2.58)	2.08 (1.72,2.45)	2.12 (1.72,2.53)	0.0225^2^
**Triglyceride [mmol/L]**	1.24 (0.94,1.67)	1.11 (0.83,1.58)	1.19 (0.90,1.64)	<0.0001^2^

1 p-value derived from Fisher's exact test.

2 p-value derived from Wilcoxon rank sum test.

The genotype and allele frequencies of the six SNPs which were included in the analysis are shown in Table 2. There was no difference in the allele frequency distribution between the GINIplus and LISAplus studies.

**Table 2:** Characteristics of the SNPs in the *FADS* gene cluster.

SNP	Alleles	N	Number of subjects with				
	(major/minor)		genotype (%)			allele (%)	
	A/a		aa	Aa	AA	a	A
rs174545	G/C	1829	211 (12%)	793 (43%)	825 (45%)	1215 (33%)	2443 (67%)
rs174546	G/A	1854	211 (11%)	804 (43%)	839 (45%)	1226 (33%)	2482 (67%)
rs174556	G/A	1849	165 (9%)	758 (41%)	926 (50%)	1088 (29%)	2610 (71%)
rs174561	A/G	1867	165 (9%)	766 (41%)	936 (50%)	1096 (29%)	2638 (71%)
rs174575	C/G	1974	139 (7%)	738 (37%)	1097 (56%)	1016 (26%)	2932 (74%)
rs3834458	T/DEL	1971	216 (11%)	855 (43%)	900 (46%)	1287 (33%)	2655 (67%)

Five of the six SNPs (rs174545, rs174546, rs174556, rs174561 and rs3834458) are in high LD with each other. For these five SNPs, the pairwise squared correlations r^2^ ranged from 0.83 to 0.99, and Lewontin's D' ranged from 0.98 to 1.

For rs174575, the linkage disequilibrium is lower. The pairwise correlation r^2^ for this SNP ranged from 0.49 to 0.66 and Lewontin's D' ranged from 0.77 to 0.96.

Median levels of total cholesterol, HDL, LDL and triglyceride concentrations, stratified by *FADS* genotype, are presented in Table 3. Homozygous minor allele carriers had lower levels of total cholesterol and LDL compared to homozygous or heterozygous major allele carriers. In contrast, triglyceride concentrations were higher in minor allele carriers compared to homozygous major allele carriers. After adjustment for multiple testing (α_corr_ = 0.025), these associations remained significant for triglyceride levels and LDL (rs174556 and rs174561).

**Table 3:** Median total cholesterol, HDL, LDL and triglyceride concentrations [mmol/L] in with 25%- and 75%-quantiles stratified by *FADS* genotype (A: major allele/ a: minor allele).

	Total cholesterol	HDL	LDL	Triglyceride
**rs174545**				
AA	4.81 (4.29,5.34)	1.27 (1.06,1.47)	2.13 (1.74,2.57)	1.14 (0.87,1.57)
Aa	4.77 (4.28,5.30)	1.23 (1.06,1.42)	2.13 (1.74,2.51)	1.23 (0.93,1.67)
aa	4.68 (4.18,5.16)	1.26 (1.09,1.43)	2.01 (1.67,2.47)	1.23 (0.92,1.69)
p-value^1^	0.1189	0.2246	0.0718	**0.0058**
**rs174546**				
AA	4.82 (4.29,5.34)	1.27 (1.07,1.47)	2.13 (1.74,2.57)	1.14 (0.88,1.57)
Aa	4.77 (4.28,5.31)	1.23 (1.06,1.42)	2.13 (1.74,2.51)	1.24 (0.94,1.68)
aa	4.66 (4.18,5.15)	1.26 (1.09,1.43)	2.01 (1.67,2.47)	1.22 (0.92,1.68)
p-value^1^	0.0793	0.1494	0.0701	**0.0078**
**rs174556**				
AA	4.83 (4.30,5.38)	1.27 (1.07,1.47)	2.14 (1.74,2.58)	1.15 (0.88,1.58)
Aa	4.76 (4.28,5.30)	1.23 (1.06,1.41)	2.12 (1.73,2.49)	1.23 (0.94,1.69)
aa	4.65 (4.18,5.08)	1.25 (1.09,1.43)	1.98 (1.65,2.38)	1.22 (0.90,1.68)
p-value^1^	0.0263	0.0842	**0.0182**	**0.0071**
**rs174561**				
AA	4.83 (4.30,5.38)	1.27 (1.07,1.47)	2.14 (1.74,2.59)	1.15 (0.88,1.58)
Aa	4.76 (4.28,5.29)	1.23 (1.06,1.42)	2.12 (1.73,2.49)	1.23 (0.94,1.69)
aa	4.65 (4.18,5.11)	1.25 (1.09,1.43)	2.00 (1.65,2.38)	1.25 (0.92,1.68)
p-value^1^	0.0288	0.1042	**0.0145**	**0.0076**
**rs174575**				
AA	4.80 (4.28,5.32)	1.26 (1.06,1.46)	2.12 (1.73,2.53)	1.15 (0.88,1.57)
Aa	4.80 (4.31,5.35)	1.23 (1.06,1.43)	2.16 (1.75,2.54)	1.25 (0.93,1.72)
aa	4.63 (4.18,5.08)	1.24 (1.04,1.41)	1.96 (1.68,2.36)	1.24 (0.98,1.70)
p-value^1^	0.0390	0.3346	0.0325	**0.0032**
**rs3834458**				
AA	4.81 (4.30,5.35)	1.26 (1.06,1.47)	2.13 (1.73,2.56)	1.15 (0.88,1.58)
Aa	4.79 (4.28,5.33)	1.23 (1.06,1.42)	2.16 (1.75,2.53)	1.24 (0.94,1.68)
aa	4.65 (4.23,5.14)	1.25 (1.06,1.43)	2.01 (1.68,2.40)	1.22 (0.94,1.69)
p-value^1^	0.0434	0.2505	0.0414	**0.0081**

1 p-value derived from Kruskal-Wallis rank sum test. Significance level after correction for multiple testing: α_corr_ = 0.025. Values reaching significance after adjustment for multiple testing are highlighted in bold.

The results of the linear regression models for total cholesterol, HDL, LDL and triglyceride concentrations, the *FADS* genotype and n-3 PUFA intake are presented in Table 4. Adjusted means ratios for total cholesterol, LDL and triglyceride concentrations and effect estimates for HDL are shown.

**Table 4:** Results of linear regression models for total cholesterol, HDL, LDL and triglyceride concentrations, *FADS* genotype (A: major allele/ a: minor allele, reference: homozygous major allele) and n-3 PUFA intake (per IQR increase, IQR (n-3 PUFA)  = 0.04 mg/MJ) adjusted for gender, study centre, age, BMI, fasting status and total dietary energy intake [MJ].

	Total cholesterol			HDL			LDL			Triglycerides		
	MR	95% CI	p-value1	Estimate	Sd	p-value1	MR	95% CI	p-value1	MR	95% CI	p-value1
**rs174545**	n = 1532			N = 1531			n = 1531			n = 1531		
n-3 PUFA	1.01	(1.00, 1.02)	0.0568	0.02	0.01	**0.0193**	1.01	(1.00, 1.03)	0.1023	0.98	(0.96, 1.00)	0.0841
Aa (ref AA)	1.00	(0.98, 1.02)	0.8311	−0.04	0.02	**0.0214**	1.01	(0.98, 1.04)	0.6525	1.06	(1.02, 1.11)	**0.0063**
aa (ref AA)	0.98	(0.95, 1.00)	0.0909	−0.01	0.02	0.6368	0.97	(0.92, 1.02)	0.1758	1.05	(0.97, 1.12)	0.2148
**rs174546**	n = 1554			n = 1553			n = 1553			n = 1553		
n-3 PUFA	1.01	(1.00, 1.02)	0.0823	0.02	0.01	**0.0147**	1.01	(1.00, 1.03)	0.1072	0.98	(0.95, 1.00)	0.0413
Aa (ref AA)	1.00	(0.98, 1.02)	0.9721	−0.04	0.02	**0.0113**	1.01	(0.97, 1.04)	0.7541	1.07	(1.02, 1.11)	**0.0048**
aa (ref AA)	0.97	(0.95, 1.00)	0.0592	−0.01	0.02	0.6663	0.97	(0.92, 1.02)	0.1777	1.03	(0.96, 1.11)	0.3621
**rs174556**	n = 1548			n = 1547			n = 1547			n = 1547		
n-3 PUFA	1.01	(1.00, 1.02)	0.0884	0.02	0.01	**0.0173**	1.01	(1.00, 1.03)	0.1075	0.98	(0.95, 1.00)	0.0363
Aa (ref AA)	1.00	(0.98, 1.01)	0.6460	−0.04	0.02	**0.0074**	0.99	(0.96, 1.03)	0.7434	1.07	(1.02, 1.12)	**0.0035**
aa (ref AA)	0.96	(0.93, 0.99)	**0.0093**	0.00	0.03	0.8902	0.94	(0.88, 0.99)	**0.0179**	1.02	(0.94, 1.10)	0.6769
**rs174561**	n = 1564			n = 1563			n = 1563			n = 1563		
n-3 PUFA	1.01	(1.00, 1.02)	0.0577	0.02	0.01	0.0306	1.01	(1.00, 1.03)	0.0790	0.98	(0.96, 1.00)	0.0533
Aa (ref AA)	1.00	(0.98, 1.01)	0.7075	−0.04	0.02	**0.0094**	1.00	(0.96, 1.03)	0.8039	1.07	(1.02, 1.11)	**0.0041**
aa (ref AA)	0.96	(0.93, 0.99)	**0.0107**	0.00	0.03	0.9700	0.94	(0.89, 0.99)	**0.0207**	1.02	(0.95, 1.11)	0.5278
**rs174575**	n = 1662			n = 1661			n = 1661			n = 1661		
n-3 PUFA	1.01	(1.00, 1.02)	0.0290	0.02	0.01	**0.0140**	1.02	(1.00, 1.03)	0.0411	0.97	(0.95, 1.00)	0.0258
Aa (ref AA)	1.01	(1.00, 1.03)	0.1070	−0.01	0.01	0.3318	1.03	(0.99, 1.06)	0.1020	1.07	(1.02, 1.11)	**0.0028**
aa (ref AA)	0.98	(0.95, 1.02)	0.2949	−0.01	0.03	0.6907	0.97	(0.91, 1.03)	0.2963	1.07	(0.99, 1.16)	0.0922
**rs3834458**	n = 1659			n = 1658			n = 1658			n = 1658		
n-3 PUFA	1.01	(1.00, 1.02)	0.0288	0.02	0.01	**0.0126**	1.02	(1.00, 1.03)	0.0422	0.97	(0.95, 1.00)	**0.0219**
Aa (ref AA)	1.00	(0.98, 1.02)	0.9479	−0.03	0.01	0.0324	1.01	(0.98, 1.04)	0.6232	1.06	(1.02, 1.11)	**0.0065**
aa (ref AA)	0.97	(0.95, 1.00)	0.0704	−0.01	0.02	0.6009	0.97	(0.92, 1.01)	0.1674	1.04	(0.98, 1.12)	0.2130

1 Significance level after correction for multiple testing: α_corr_ = 0.025. Values reaching significance after adjustment for multiple testing are highlighted in bold.

The p-values describing the association between n-3 PUFA intake and elevated concentrations of total cholesterol [MR = 1.01 for all six SNPs (p-value ranging from 0.0288 to 0.0884)], LDL [MR from 1.01 (p = 0.1075) to 1.02 (p = 0.0411)], HDL [β = 0.02 for all six SNPs (p-value between 0.0126 and 0.0306)] and reduced triglyceride levels [MR between 0.97 (p = 0.0219) and 0.98 (p = 0.0841)] were statistically significant. However, after correcting for multiple testing, only five (out of six) SNPs for HDL and triglyceride levels and rs3834458 remained significant.

Additional analyses showed similar results for the n-3 PUFAs ALA, EPA, DPA and DHA, which were combined into the total dietary n-3 PUFA intake (data not shown).

There was no association between dietary n-6 PUFA intake and lipid levels (data not shown).

Homozygous minor allele carriers had decreased levels of total cholesterol [MR ranging from 0.96 (p = 0.0093) to 0.98 (p = 0.2949)] and LDL [MR between 0.94 (p = 0.0179) and 0.97 (p = 0.2963)] compared to homozygous major allele carriers. After correcting for multiple testing (α_corr_ = 0.025), the association remained significant for rs174556 and rs174561 and total cholesterol and LDL.

HDL concentrations were reduced in carriers of the heterozygous genotype compared to homozygous major allele carriers [β between −0.04 (p = 0.0074) to −0.01 (p = 0.3318)]. These associations remained significant for four SNPs after correction for multiple testing.

Individuals carrying the heterozygous genotype showed significantly increased triglyceride levels compared to homozygous major allele carriers [MR ranging from 1.06 (p = 0.0065) to 1.07 (p = 0.0028)]. These associations remained significant after correcting for multiple testing. Although homozygous minor allele carriers also showed increased triglyceride levels, these effects did not reach statistical significance. Additional analyses restricted to fasting blood samples did not show substantially different results and the magnitude of the association between *FADS* genotype, n-3 PUFA and lipid concentration was similar. However, this result did not reach statistical significance, likely because of the reduced sample size (Table S1).

For each model, including the *FADS* SNP increased the percentage of explained variance compared to the model without any SNPs (Table S2). The maximal increase in the explained variance was 0.71% for the total cholesterol model (1.98% to 2.69%), 1.28% for the LDL model (from 4.21% to 5.49%), 0.96% for the HDL model (from 7.83% to 8.79%) and 0.48% for the triglycerides model (from 12.38% to 12.86%). Additional analyses stratified by study (GINIplus and LISAplus) showed similar results, although again, the lack of statistical significance is likely attributable to the reduced sample size (Figure S1).

There was no significant interaction between n-3 PUFA intake and *FADS1 FADS2* genotype for any of the tested lipid concentrations (data not shown).

## Discussion

The present study investigated the association between n-3 PUFA intake and *FADS* genotype with total cholesterol, HDL, LDL and triglyceride concentrations in 10-year-old children from the GINIplus and LISAplus birth cohort studies.

Although not all associations were statistically significant after adjustment for multiple testing, in these children, a higher n-3 PUFA intake was associated with higher total cholesterol, HDL and LDL and lower triglyceride levels. These associations remained significant after adjustment for multiple testing for HDL (five out of six tested SNPs) and triglyceride concentrations (rs3834458).

Minor alleles of *FADS1* and *FADS2* SNPs were significantly associated with higher levels of triglycerides and lower levels of total cholesterol, HDL, and LDL levels. However, not all of these associations reached statistical significance after correcting for multiple testing.

Generally, our results on trends in children are in line with previously published GWA studies that report an association between the minor allele of the tested *FADS* variant with lower total cholesterol [11], LDL [11]–[13], HDL [13], [14] and higher triglyceride [14], [15] concentrations in adults. Although the sample size of our cohort is relatively large, failure to reach statistical significance for all SNPs after correcting for multiple testing may be due to a lack of statistical power and small effect sizes. Nevertheless, our effect sizes for total cholesterol and triglycerides are comparable to those reported for adults [11], [14]_._ Bokor et al. [24] investigated the association between *FADS* haplotypes and lipid levels in adolescents, and reported no associations between any of the haplotypes and total cholesterol, HDL or LDL. However, the haplotype carrying the minor allele of rs174546 was significantly associated with higher triglyceride levels and the effect size reported is similar to that observed in our study.

The lack of statistical significance with respect to triglyceride levels in homozygous minor allele carriers may be due to the small size of this group (7% to 12% of the complete sample).

The inclusion of the *FADS* SNPs to the model lead to an increase of explained variance ranging from 0.48% for the triglyceride model to 1.28% for the LDL model, which showed a similar level as reported by several studies [25].

Lipid levels in children are determinants for cardiovascular diseases in adulthood [5]–[7]. Analysis of the underlying causes for disturbances in lipid metabolism during childhood can contribute to the prevention of cardiovascular diseases later in life. Our study suggests that the *FADS1 FADS2* gene cluster may influence lipid levels in early life.

The underlying causal biological mechanism between an individual's *FADS* genotype and their lipid concentrations is not entirely clear. It is likely that the composition of polyunsaturated fatty acids in human tissues, which has been shown to be highly associated with the *FADS* genotype [17], is the direct link between the observed associations. Tanaka et al. [26] presumed that higher concentrations of the precursor fatty acids in minor allele carriers may result in increased membrane fluidity, and thus, in lower LDL. In addition to altered membrane fluidity, differential concentrations of long-chain PUFAs (LC-PUFAs) may lead to a change in the activation of transcription factors such as peroxisome proliferator activating receptor alpha (PPARA). Endogenous LC-PUFAs are natural ligands of PPARA [27], whose activation has been shown to influence the expression of apo-lipoproteins (e.g. ApoAI, Apo-AII, and ApoCIII) and enzymes (lipoprotein lipase) that are involved in the metabolism of lipoprotein particles [28]–[31].

The effect of *FADS* genotypes on fatty acid levels, which are also influenced by nutrition, leads to further interesting questions. One of which is whether there is a concerted interaction effect between *FADS* genotypes and PUFA intake on lipid levels. To date, few studies have dealt with this issue. Lu et al. [32] reported an association between *FADS* genotype and lipid levels, but only in groups with a high n-3 or n-6 PUFA intake. This result suggests an interaction between genotype and fatty acid intake, although a complete interaction analysis was not performed in this study. In our study, a higher intake of n-3 PUFA was associated with higher total cholesterol, HDL and LDL levels and lower triglyceride concentrations, although these effects were not significant after adjustment for multiple testing. We did not find any association between lipid levels, *FADS* genotype and dietary n-6 PUFA intake (data not shown). In our interaction analysis, we did not find a modification of the effect of n-3 PUFA intake on lipid concentrations in blood by the *FADS* genotype.

Additionally, the number of multiple comparisons has to be considered. In our manuscript, 24 hypotheses are investigated (6 SNPs and 4 traits). A correction for multiple testing is necessary, if several independent hypotheses are tested simultaneously. In the present manuscript, neither the SNPs, nor traits are independent. A more stringent approach, which would account for the number of outcome variables, would lead to a corrected alpha level of 0.0063. Taking this alpha level as a basis, the association between individuals carrying the heterozygous genotype and triglyceride levels compared to homozygous major allele carriers remains significant for five out of six SNPs. However, the reported associations with each of the selected six SNPs with traits were similar, possibly due to high correlation. A chance finding would be obvious, if just one of the tested SNPs were highly statistical significant, where the others were not. This is not the case in our analyses. Therefore, we do not consider these results as chance finding.

Nevertheless, our results suggest that there is an effect of n-3 PUFA intake as well as an effect of *FADS* genotype on lipid levels, although these effects do not interact with each other. It is known, that the conversion rate from dietary PUFA intake to longer chain metabolites is depending on the *FADS* genotype [17]. Therefore, it might be possible that dietary n-3 PUFA intake as well as the *FADS* genotype have a linear influence on the endogenous PUFA levels. This would be in line with the results presented by Moltó-Puigmartí et al. [22]. They reported lower DHA proportions in plasma phospholipids in women carrying the homozygous minor allele, but the DHA proportions increased with higher intake of fatty fish to a similar extent for all genotypes.

To our knowledge, this is the first study which has examined the complex associations between genetics, diet, and lipid levels in 10 year old children.

In addition to its strength, our study also faces some limitations which must be considered. Although lipid levels were measured from blood samples, the dietary fatty acid intake was assessed by a FFQ. The FFQ used in the present study measured dietary intake over the past 12 months and was validated for the dietary n-3 PUFA intake. Alternatively, it might be interesting to examine the association of *FADS* genes, lipid concentrations and measured fatty acid blood levels. A further limitation of our study is the low proportion of fasting blood samples (18.4%). The presented results were adjusted for fasting status. Additionally, the magnitude of the association between *FADS* genotype, n-3 PUFA and lipid concentration was similar when the sample was restricted to those with information on fasting blood, although statistical significance was lacking due to a reduced sample size (Results for triglyceride levels are presented in Table S1).

In order to increase the power, the two independent studies GINIplus and LISAplus were analyzed together. Additional analyses stratified for the GINIplus and LISAplus studies showed consistent results (Figure S1), except for LDL, although significance was missed due to the reduced sample size. This investigation addressed a complex hypothesis and is based on a specific data situation: Dietary fatty acid intake was assessed using a FFQ, which was especially developed and validated for dietary fatty acid intake in this specific study population [33], blood lipid levels of total cholesterol, HDL, LDL and triglycerides were measured and genotyping of six variants of the *FADS1 FADS2* gene cluster was performed. Nevertheless, the results in the two independent studies GINIplus and LISAplus, which are based on the same methodology, are similar and our results are comparable to those reported for adults.

Our study suggests that the *FADS1 FADS2* gene cluster may affect lipid levels already in childhood. Although the explained variance is low, and can therefore not be used for prevention or prediction purposes, these results underline the hypothesis that there is a causal association between dietary n-3 PUFA intake and lipid levels in children and may help to identify the causal biological mechanism.

However, further studies are needed to investigate the long-term effects of the impact of dietary intervention on the development of cardiovascular diseases, while considering the influence of the *FADS* gene cluster.

## Materials and Methods

### Study population

Data from two ongoing German birth cohort studies were included in this investigation: the German LISAplus (Life-style Related Factors on the Immune System and the Development of Allergies in Childhood PLUS the influence of traffic emissions and genetics) and GINIplus (German Infant Nutritional Intervention PLUS environmental and genetic influences on allergy development) studies. LISAplus is a population based birth cohort study in which a total of 3097 neonates were recruited between 1997 and 1999 from the German cities of Munich, Leipzig, Wesel and Bad Honnef. The participants were not pre-selected based on family history of allergic diseases [34]. A total of 5991 mothers and their newborns were recruited from Munich and Wesel into the GINIplus study between September 1995 and June 1998. Infants with at least one allergic parent and/or sibling were allocated to the interventional study arm which investigated the effect of different hydrolysed formulas consumed during the first year of life on the development of allergies [35]. All children without a family history of allergic diseases and children whose parents did not give consent for the randomized clinical trial were allocated to the non-interventional arm. Given that the entire current GINIplus study is composed of both the interventional and non-interventional arm, this cohort is also population-based. Detailed descriptions of the LISAplus and GINIplus studies have been published elsewhere [34]–[36]).

In both studies only individuals with Caucasian German descent were included.

For this analysis, only data from the 10 year follow-up is used. During this follow-up, blood samples were collected and in a subset of almost 20% of the children fasting blood samples could be collected.

For both studies, approval by the local Ethics Committees (Bavarian Board of Physicians, University of Leipzig, Board of Physicians of North-Rhine-Westphalia) and written consent from participant's families were obtained.

### Dietary n-3 PUFA intake

A food frequency questionnaire (FFQ) was developed to measure a child's usual food and nutrient intake during one year, and more specifically, to estimate energy, fatty acid and antioxidant intake at 10 years of age. The FFQ comprised a list of 82 food items accompanied by several questions about the preferred fat and energy content of products, preparation methods, diet and food preferences, buying habits and dietary supplement use. The consumption frequencies and portion size estimates were converted to average consumption in grams per day and linked to the German Nutrient Data Base, version II.3.1 [37]. The design of the FFQ, including the selection of the food item list, validation, and the calculation of food and nutrient intake is described in more detail by Stiegler et al. [33].

The intake of n-3 PUFA was calculated by summing a child's daily intake of α-linolenic acid (ALA, 18: 3n-3), eicosapentaenoic acid (EPA, 20: 5n-3), docosapentaenoic acid (DPA, 22: 5n-3) and docosahexaenoic acid (DHA, 22: 6n-3).

### Genotyping

Six single nucleotide polymorphisms (SNPs) in the *FADS1 FADS2* gene cluster (rs174545, rs174546, rs174556, rs174561, rs174575 and rs3834458) were genotyped. Five of these variants (rs174545, rs174546, rs174556, rs174561 and rs3834458) are in strong linkage disequilibrium (LD) with each other (r^2^>0.7, D'>0.9) [17], and were selected based on previous publications in adult populations [17], [38]. Additionally, we included the rs174575 SNP in order to obtain a better coverage of the *FADS* gene cluster. This SNP was selected based on a previous publication in children [39]. By applying the tagger server program (http://www.broadinstitute.org/mpg/tagger/) in combination with HapMap (http://hapmap.ncbi.nlm.nih.gov/), we were able to tag 27 additional SNPs between base pair positions 61234329 and 61372379 in the *FADS1 FADS2* gene cluster using three of our original SNPs (rs174545, rs174546 and rs174556). The efficiency was 10.7 fold, however of these new 27 SNPs, rs174561 and rs3834458 could not be used in the analysis as these are not included in the HapMap database. Genotyping of SNPs was conducted using the iPLEX (Sequenom, San Diego, CA, USA) method by means of matrix assisted laser desorption ionization-time of flight mass spectrometry (MALDI-TOF MS, Mass Array; Sequenom) in one laboratory, according to the manufacturer’s instructions. Standard genotyping quality control included 10% duplicate and negative samples. The genotyping discordance rate was below 0.3%.

### Measurement of lipids

The measurement of serum lipids and lipoproteins was performed using homogenous enzymatic colorimetric methods according to the manufactures instructions (Roche Diagnostics GmbH Mannheim). All parameters and controls were analysed on a Modular Analytics System from Roche Diagnostics GmbH Mannheim.

External controls were used in accordance with the guidelines of the German Society of Clinical Chemistry and Laboratory Medicine.

### Statistical analysis

Because of the skewed distribution, total cholesterol, LDL and triglyceride concentrations were naturally log-transformed. Afterwards, linear regression modelling was used to assess the association between log-transformed total cholesterol, HDL, log-transformed LDL and log-transformed triglyceride concentrations, n-3 PUFA intake and *FADS* genotype. Therefore, for HDL, the regression coefficient β (Estimate) with standard deviation (Sd) is shown.

For total cholesterol, LDL and triglyceride, the results are presented as means ratio (MR) with 95% confidence interval (95% CI). The MR can be easily calculated for lognormal distributed variables and is derived by applying the exponential function on the regression coefficient β (i.e. MR = exp (β)). It describes the ratio of the mean of the outcome variable in one group compared to the mean of the outcome variable in the reference group. The MR can be interpreted as percentage change in the mean of the outcome variable in one group compared to the reference group adjusted for confounder variables.

The dietary n-3 PUFA intake was included in the regression analyses as nutrient density (n-3 PUFA intake divided by total energy intake). Additionally, the results were adjusted for total energy intake [40]. In order to compare the effect size of high (75% quantile) and low (25% quantile) dietary n-3 PUFA intake, the influence of n-3 PUFA intake is given per interquartile range increase (IQR (n-3 PUFA)  = 0.04 mg/MJ).

All regression models were adjusted for gender, study centre (Munich, Wesel, Leipzig and Bad Honnef), age, BMI at 10 years of age and fasting status.

Moreover, in additional analyses, an interaction between n-3 PUFA and *FADS* genotype (reference: homozygous major allele), was included in the linear regression models in order to test whether the effect of dietary n-3 PUFA intake is modified by *FADS* variants.

Statistical significance was defined by a two-sided alpha level of 5%. We corrected for multiple testing according to Nyholt [41]. In brief, this method takes the correlation pattern between the SNPs into account and reduces the number of variables in a set to the effective number of variables and provides thereby an estimate of the number of independent tests.

The alpha level is divided by the number of effective loci (which was computed as two, based on the number of effective loci of the six SNPs in the *FADS* gene cluster), which yields a corrected two-sided alpha level of 0.025 (5%/2 = 2.5%).

Differences between the GINIplus and LISAplus studies were tested using Fisher's exact test or Wilcoxon rank sum test. The association between lipid concentrations and *FADS* genotype was tested using Kruskal-Wallis rank sum test, a nonparametric method to test whether the median of the lipid concentrations is different between the *FADS* genotypes.

Statistical analysis was performed using the statistical software R, version 2.13.1 (http://www.R-project.org) [42].

## Supporting Information

Figure S1 **Results of linear regression models on total cholesterol, HDL, LDL and triglycerides stratified for the GINIplus and LISAplus studies.** Presented are means ratios (total cholesterol, LDL and triglycerides) and effect estimates (HDL) of *FADS* genotype (A: major allele/ a: minor allele, reference: homozygous major allele) and n-3 PUFA intake (per IQR increase, IQR (n-3 PUFA)  = 0.04 mg/MJ). All models are adjusted for gender, study centre, age, BMI and total dietary energy intake [MJ]. a) Total cholesterol b) HDL c) LDL d) Triglycerides
(DOC)

Table S1 **Results of linear regression models restricted to fasting blood samples for triglyceride concentrations,** ***FADS*** **genotype (A: major allele/ a: minor allele, reference: homozygous major allele) and n-3 PUFA intake (per IQR increase, IQR (n-3 PUFA)  = 0.04 mg/MJ) adjusted for gender, study centre, age, BMI and total dietary energy intake [MJ].**
(DOC)

Table S2 **Percentage of variance explained in the models without and with the** ***FADS*** **variants.**
(DOC)
