# Supplementary figures and images for: FADS1 FADS2 Gene Cluster, PUFA Intake and Blood Lipids in Children: Results from the GINIplus and LISAplus Studies
Source: PLoS One. 2012 May 21;7(5):e37780. doi: 10.1371/journal.pone.0037780 (PMC3357401; doi:10.1371/journal.pone.0037780)

**Figure S1**

a) Total cholesterol


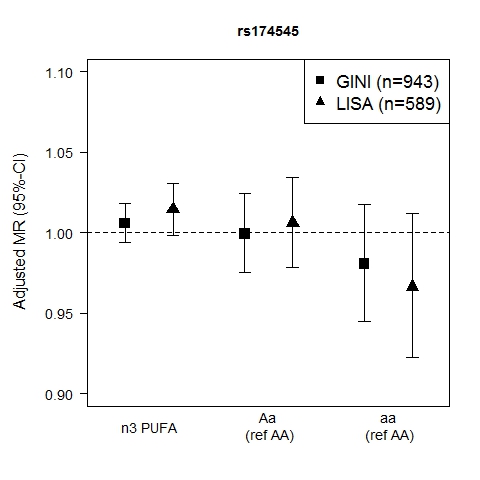

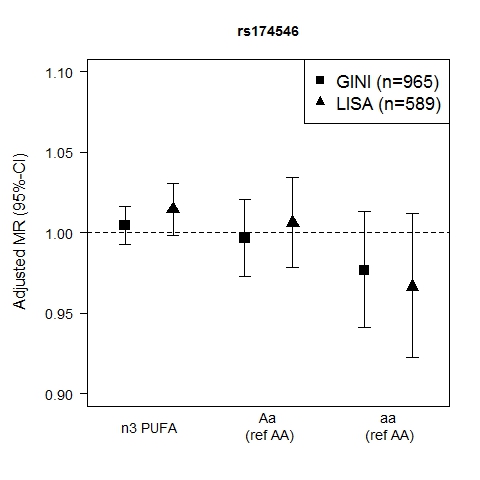

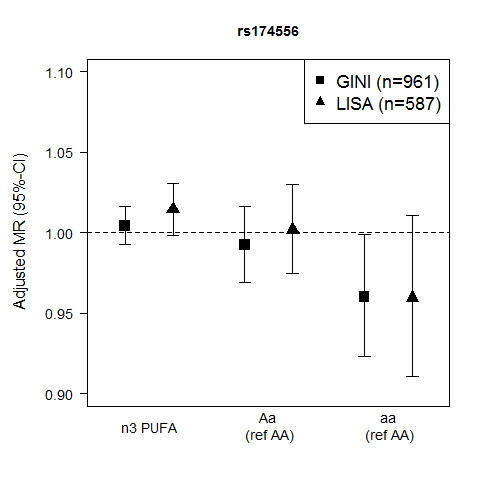

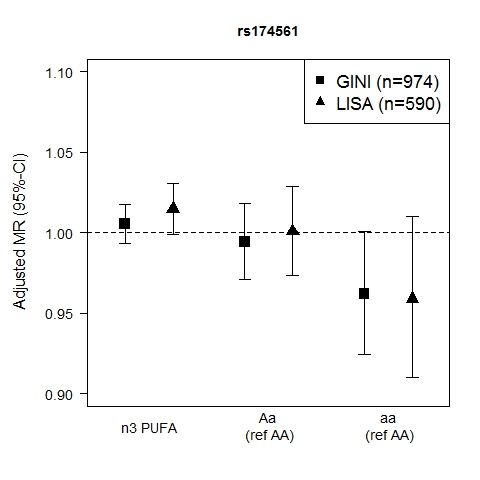

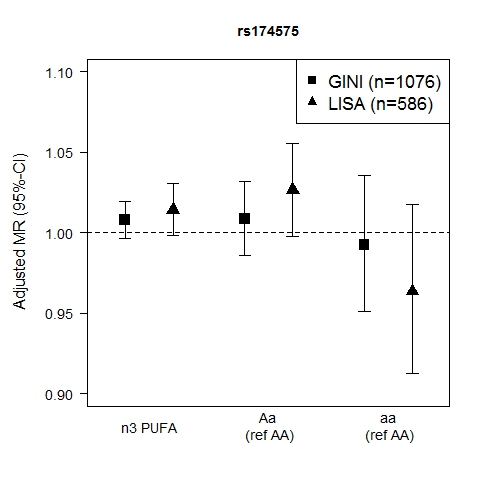

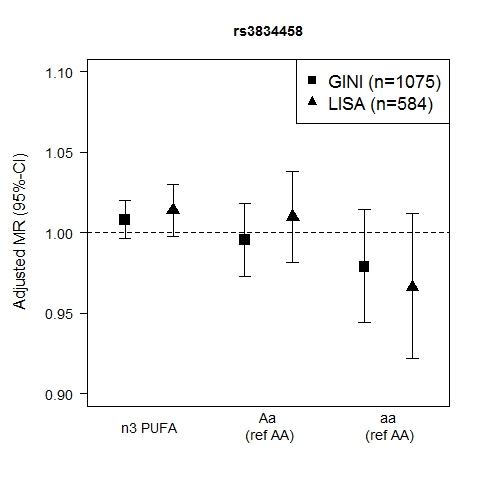


b) HDL


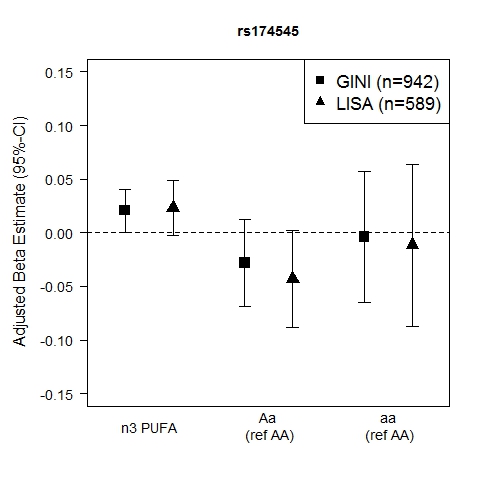

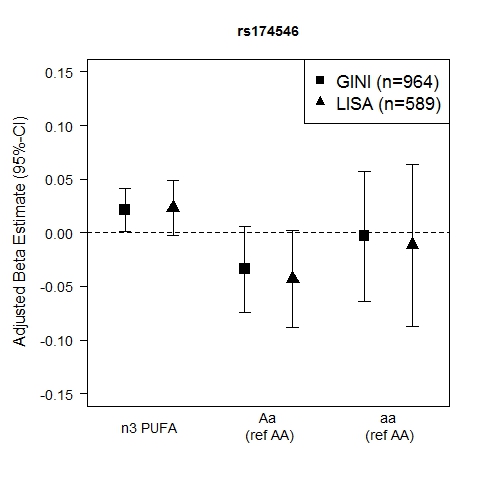

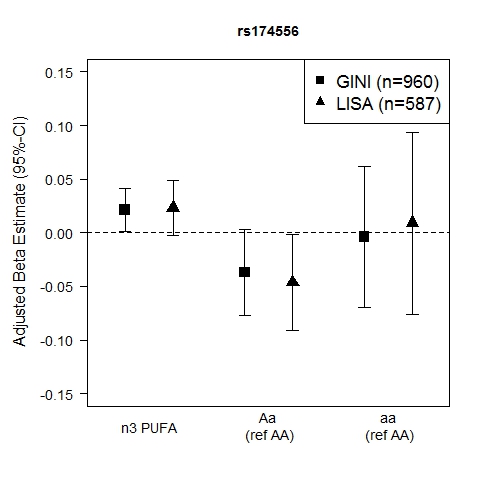

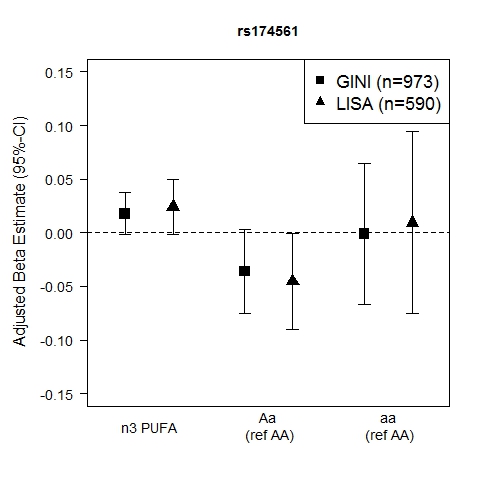

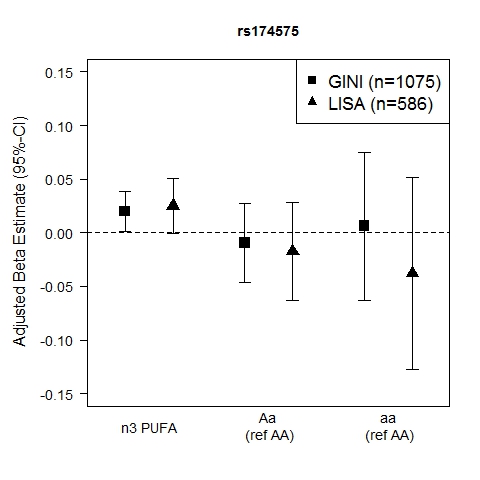

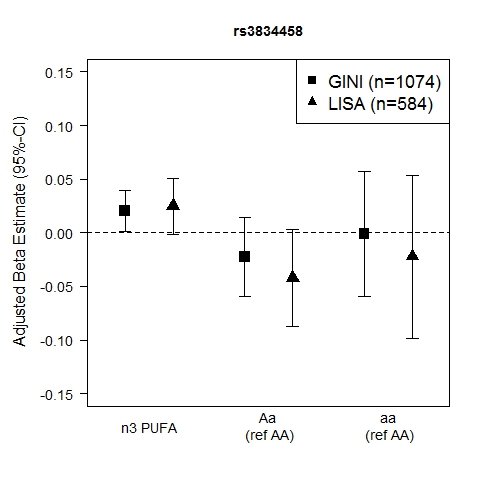


c) LDL


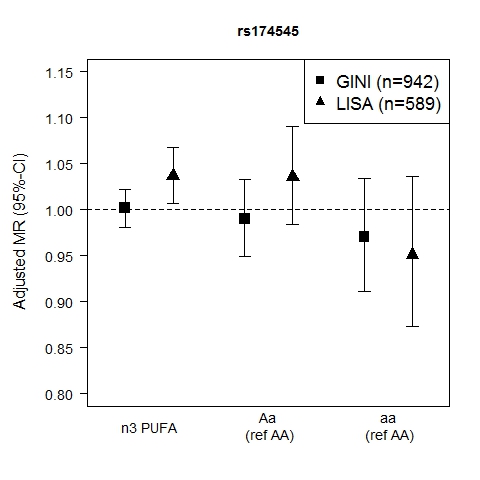

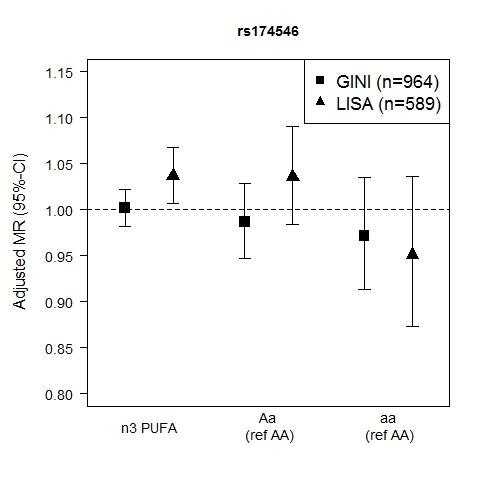

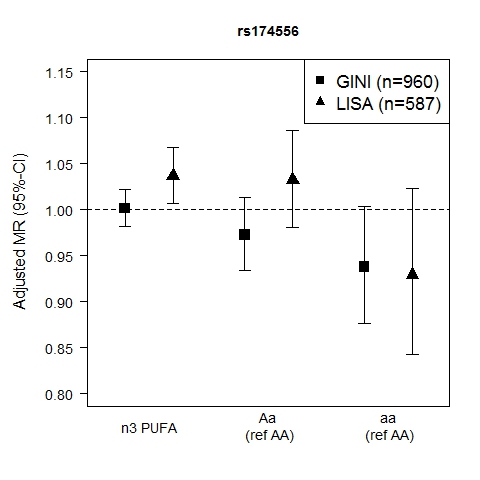

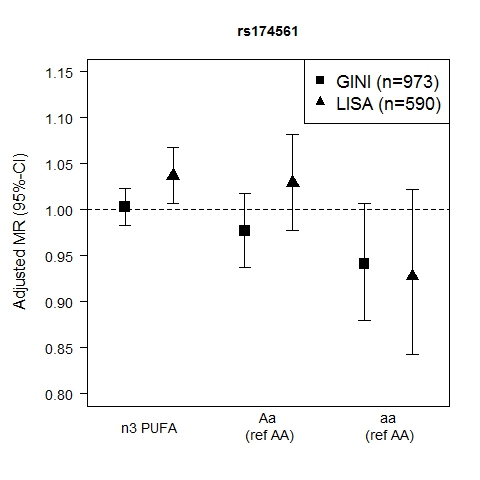

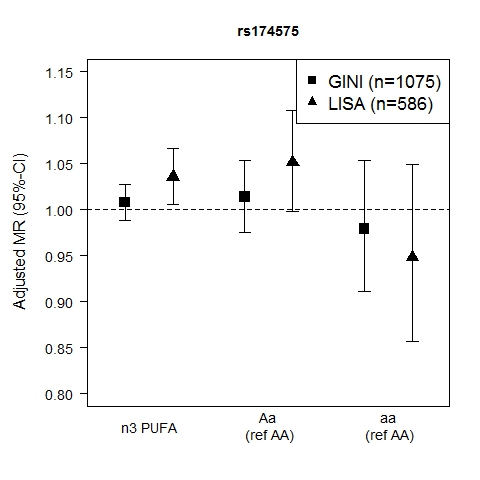

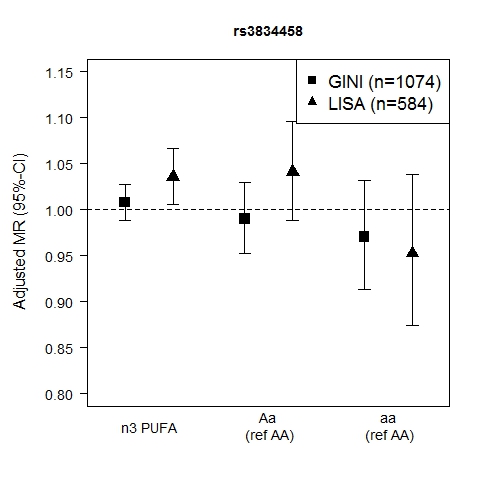


d) Triglycerides


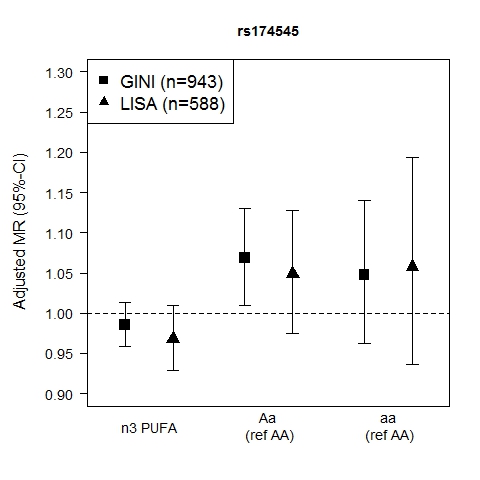

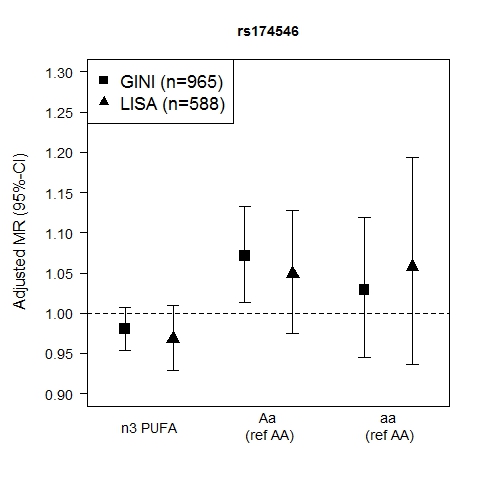

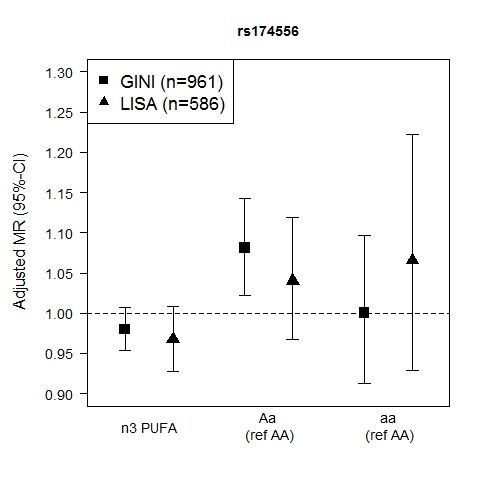

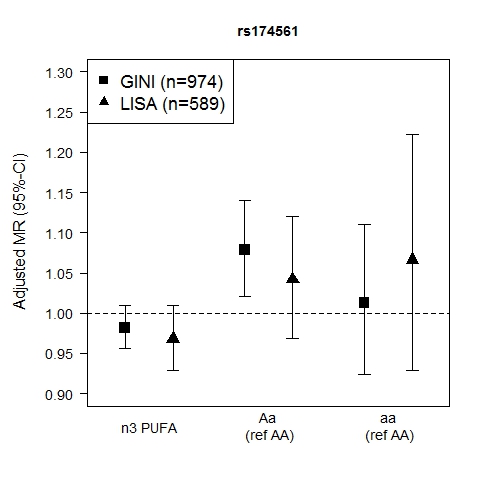

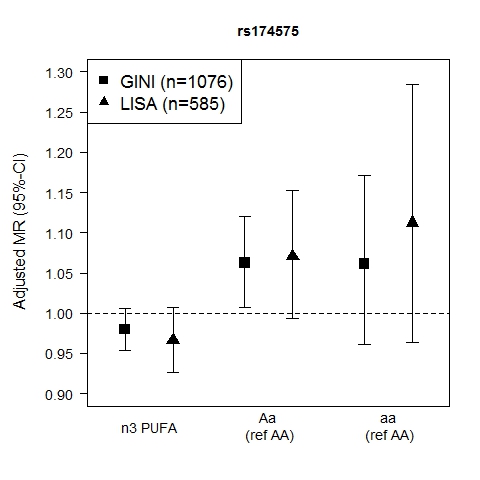

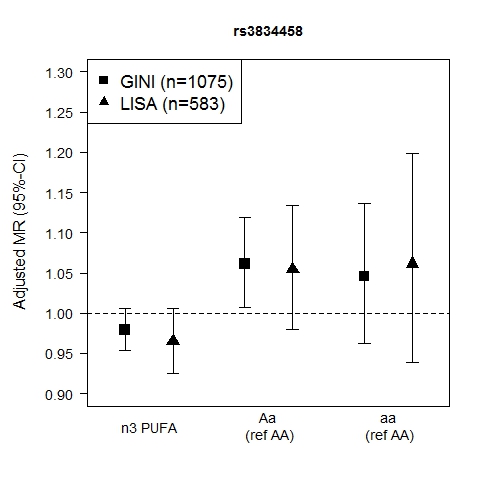

Supplement: Figure S1 — Results of linear regression models on total cholesterol, HDL, LDL and triglycerides stratified for the GINIplus and LISAplus studies. Presented are means ratios (total cholesterol, LDL and triglycerides) and effect estimates (HDL) of FADS genotype (A: major allele/ a: minor allele, reference: homozygous major allele) and n-3 PUFA intake (per IQR increase, IQR (n-3 PUFA) = 0.04 mg/MJ). All models are adjusted for gender, study centre, age, BMI and total dietary energy intake [MJ]. a) Total cholesterol b) HDL c) LDL d) Triglycerides (DOC) [file pone.0037780.s001.doc]
