# Supplementary material for: FADS1 FADS2 Gene Cluster, PUFA Intake and Blood Lipids in Children: Results from the GINIplus and LISAplus Studies
Source: PLoS One. 2012 May 21;7(5):e37780. doi: 10.1371/journal.pone.0037780 (PMC3357401; doi:10.1371/journal.pone.0037780)
Supplement: Table S1 — Results of linear regression models restricted to fasting blood samples for triglyceride concentrations, FADS genotype (A: major allele/ a: minor allele, reference: homozygous major allele) and n-3 PUFA intake (per IQR increase, IQR (n-3 PUFA) = 0.04 mg/MJ) adjusted for gender, study centre, age, BMI and total dietary energy intake [MJ]. (DOC) [file pone.0037780.s002.doc]

Table S1

|  | **MR** | **95% CI** | **p-value** |
| --- | --- | --- | --- |
| **rs174545** | n=293 |  |  |
| n-3 PUFA | 0.97 | (0.94, 1.01) | 0.1573 |
| Aa | 1.02 | (0.93, 1.11) | 0.7443 |
| aa | 1.09 | (0.93, 1.27) | 0.2813 |
| **rs174546** | n=296 |  |  |
| n-3 PUFA | 0.97 | (0.93, 1.01) | 0.1486 |
| Aa | 1.02 | (0.94, 1.12) | 0.6004 |
| aa | 1.05 | (0.91, 1.23) | 0.4927 |
| **rs174556** | n=296 |  |  |
| n-3 PUFA | 0.97 | (0.93, 1.01) | 0.1429 |
| Aa | 1.02 | (0.93, 1.12) | 0.6167 |
| aa | 1.04 | (0.88, 1.23) | 0.6426 |
| **rs174561** | n=298 |  |  |
| n-3 PUFA | 0.97 | (0.93, 1.01) | 0.1310 |
| Aa | 1.02 | (0.93, 1.12) | 0.6457 |
| aa | 1.04 | (0.88, 1.23) | 0.6504 |
| **rs174575** | n=316 |  |  |
| n-3 PUFA | 0.97 | (0.94, 1.01) | 0.1342 |
| Aa | 0.99 | (0.91, 1.08) | 0.8606 |
| aa | 1.02 | (0.86, 1.21) | 0.7950 |
| **rs3834458** | n=315 |  |  |
| n-3 PUFA | 0.97 | (0.94, 1.01) | 0.1331 |
| Aa | 1.03 | (0.94, 1.12) | 0.5267 |
| aa | 1.06 | (0.92, 1.23) | 0.4059 |
