# Supplementary material for: FADS1 FADS2 Gene Cluster, PUFA Intake and Blood Lipids in Children: Results from the GINIplus and LISAplus Studies
Source: PLoS One. 2012 May 21;7(5):e37780. doi: 10.1371/journal.pone.0037780 (PMC3357401; doi:10.1371/journal.pone.0037780)
Supplement: Table S2 — Percentage of variance explained in the models without and with the FADS variants. (DOC) [file pone.0037780.s003.doc]

Table S2

|  | **Total cholesterol** | **LDL** | **HDL** | **Triglycerides** |
| --- | --- | --- | --- | --- |
| **Without SNP** | 1.98% | 4.21% | 7.83% | 12.38% |
| **rs174545** | 2.38% | 5.26% | 8.16% | 12.40% |
| **rs174546** | 2.44% | 5.30% | 8.45% | 12.72% |
| **rs174556** | 2.53% | 5.43% | 8.56% | 12.86% |
| **rs174561** | 2.69% | 5.49% | 8.38% | 12.47% |
| **rs174575** | 2.66% | 5.19% | 8.59% | 12.54% |
| **rs3834458** | 2.60% | 5.09% | 8.79% | 12.44% |
| **Max. difference** | 0.71% | 1.28% | 0.96% | 0.48% |
